# Association of deposition of tau and amyloid‐β proteins with structural connectivity changes in cognitively normal older adults and Alzheimer’s disease spectrum patients

**DOI:** 10.1002/brb3.1145

**Published:** 2018-10-24

**Authors:** Yoko Shigemoto, Daichi Sone, Norihide Maikusa, Nobuyuki Okamura, Shozo Furumoto, Yukitsuka Kudo, Masayo Ogawa, Harumasa Takano, Yuma Yokoi, Masuhiro Sakata, Tadashi Tsukamoto, Koichi Kato, Noriko Sato, Hiroshi Matsuda

**Affiliations:** ^1^ Integrative Brain Imaging Center National Center of Neurology and Psychiatry Tokyo Japan; ^2^ Department of Radiology National Center of Neurology and Psychiatry Tokyo Japan; ^3^ Department of Psychiatry National Center of Neurology and Psychiatry Tokyo Japan; ^4^ Division of Pharmacology, Faculty of Medicine Tohoku Medical and Pharmaceutical University Sendai Japan; ^5^ Department of Geriatric and Gerontology, Institute of Development, Aging and Cancer Tohoku University Sendai Japan; ^6^ Division of Radiopharmaceutical Chemistry, Cyclotron and Radioisotope Center Tohoku University Sendai Japan; ^7^ Department of Neurology National Center of Neurology and Psychiatry Tokyo Japan

**Keywords:** Alzheimer’s disease, diffusion tensor imaging, structural network, tau PET

## Abstract

**Introduction:**

Alzheimer's disease (AD) is characterized by accumulation of extracellular amyloid‐β and intracellular tau neurofibrillary tangles. The recent advent of tau positron emission tomography (PET) has enabled in vivo assessment of tau pathology. The aim of this study was to explore whether tau deposition influences the structural connectivity in amyloid‐negative and amyloid‐positive groups, and further explore the difference between the groups.

**Methods:**

We investigated 18 patients with amnestic mild cognitive impairment/mild AD (AD‐spectrum group) and 35 cognitively normal older adults (CN group) using diffusion MRI, amyloid, and tau PET imaging. Diffusion connectometry was performed to identify white matter pathways correlated with each of the six variables of tau deposition in the bilateral hippocampi, temporal lobes, posterior and anterior cingulate cortices, precunei, orbitofrontal lobes, and entire cerebrum.

**Results:**

The CN group showed increased connectivity along with an increased tau deposition in the bilateral hippocampi, temporal lobes, and entire cerebrum, whereas the AD‐spectrum group showed decreased connectivity in the bilateral hippocampi, temporal lobes, anterior and posterior cingulate cortices, precunei, and entire cerebrum.

**Conclusion:**

These findings suggest that tau deposition in the CN group seems to induce a compensatory response against early neuronal injury or chronic inflammation associated with normal aging, whereas the coexistence of amyloid and tau in the AD‐spectrum group seems to outweigh the compensatory response leading to decreased connectivity, suggesting that amyloid plays a crucial role in alternating structural connectivity.

## 1 INTRODUCTION

Alzheimer's disease (AD) is the most common type of progressive degenerative dementia. AD is characterized by abnormal accumulation of misfolded extracellular amyloid‐β (Aβ) and intracellular neurofibrillary tangles (NFTs) of tau proteins, which are associated with synaptic disruption and subsequent neuronal death (Braak & Braak, 1991; Terry et al., 1991; Wenk, 2003). Although ^18^F‐THK5351 was originally designed to detect tau aggregates, recent studies have clarified the existence of off‐target binding of ^18^F‐THK5351 to monoamine oxidase B (MAO‐B; Harada et al., 2018; Ishiki et al., 2018; Ng et al., 2017). Thus, ^18^F‐THK5351 retention reflects the combination of tau pathology and MAO‐B‐positive astrogliosis (Harada et al., 2018; Ishiki et al., 2018), and considered the promising biomarker for detecting neuroinflammation in the brain (Okamura et al., 2018).

Because Aβ and NFTs are associated with local synaptic disruption, AD is suggested to be a dysconnectivity syndrome characterized by abnormalities in the brain network (Arendt, 2009; Blennow, Bogdanovic, Alafuzoff, Ekman, & Davidsson, 1996; Delbeuck, Linden, & Collette, 2003; Pievani, Haan, Wu, Seeley, & Frisoni, 2011). Recent advances in neuroimaging have enabled investigation of brain networks using functional and structural magnetic resonance imaging (MRI) including diffusion tensor imaging (DTI; Achard & Bullmore, 2007; Gong et al., 2009; Iturria‐Medina, Sotero, Canales‐Rodríguez, Alemán‐Gómez, & Melie‐García, 2008). Studies using resting‐state functional MRI have revealed connectivity changes associated with tau deposition in preclinical AD (Schultz et al., 2017; Sepulcre et al., 2017). Schultz et al. (2017) demonstrated increased connectivity with low neocortical tau deposition but decreased connectivity with elevated tau deposition, raising the possibility that increased connectivity might be a compensatory response. However, it is still controversial whether connectivity as defined across neuroimaging modalities measures the same underlying construct (Gong, He, Chen, & Evans, 2012; Honey et al., 2009; Honey, Kötter, Breakspear, & Sporns, 2007). Jacobs et al. (2018) recently reported the association between structural connectivity and tau accumulation in preclinical AD. Interestingly, tau accumulation in the posterior cingulate cortex is associated with decreased connectivity of the hippocampal cingulum bundle in amyloid‐positive individuals but not in amyloid‐negative individuals. This finding indicates that amyloid plays a crucial part in alternating structural connectivity associated with tau deposition.

Diffusion connectometry compares the density of diffusion spins, which is different from diffusivity measurements such as fractional anisotropy, mean diffusivity, and radial diffusivity (Yeh, Badre, & Verstynen, 2016). Recently, this method has gained much attention (Delaparte et al., 2017; Olvet et al., 2016; Wen et al., 2016), because it overcame the problem of conventional DTI analysis, which was affected by partial volume effects or crossing fibers (Yeh, Tang, & Tseng, 2013). Diffusion connectometry first measures the degree of connectivity between adjacent voxels within a white matter fiber comparing the density of diffusion spins. It then tracks only the consecutive fiber segment that shows significant positive and negative relations with the study variables. To our knowledge, no study has evaluated structural connectivity in AD using diffusion connectometry.

Here, we hypothesized that the coexistence of amyloid plays a crucial role in alternating structural connectivity associated with tau in a similar manner as described in the study by Jacobs et al. (2018). We tested this hypothesis using automated diffusion connectometry analysis between an amyloid‐negative cognitively healthy group and an amyloid‐positive AD‐spectrum group.

## 2 MATERIALS AND METHODS

### 2.1 Participants

Participants were 35 cognitively normal older adults (CN group) and 18 patients with amnestic mild cognitive impairment (aMCI)/probable AD dementia (aMCI = 5, mild AD = 13). Participants underwent structural MRI, ^18^F‐THK5351, and ^11^C‐Pittsburgh compound B (PiB) positron emission tomography (PET) imaging from June 2015 to January 2017. All participants underwent medical and neurological examinations, and none had medical or neurological disorders that might contribute to cognitive dysfunction. Each participant underwent cognitive evaluations using the following instruments: Mini‐Mental State Examination (MMSE), Clinical Dementia Rating (CDR), Wechsler Memory Scale‐Revised Logical Memory II (WMSR LM‐II), and the Japanese version of the Montreal Cognitive Assessment (MoCA‐J). The MCI patients (*n* = 5) fulfilled the criteria for amnestic MCI established by Petersen (2004). Clinical diagnoses of probable AD (*n* = 13) were based on the clinical criteria outlined in the National Institute on Aging–Alzheimer's Association guidelines (McKhann et al., 2011). Due to the smaller numbers of aMCI and mild AD dementia patients compared with the CN group, we performed the analyses with a combined aMCI/mild AD (AD‐spectrum) group.

All participants gave written informed consent to participate in the study, which was approved by the institutional ethics committee at the National Center of Neurology and Psychiatry.

### 2.2 Image acquisition

All participants underwent MRI scans with a 3‐T MRI system (Verio; Siemens, Erlangen, Germany) using a 32‐channel head coil. High‐spatial‐resolution, 3D sagittal T1‐weighted MPRAGE (magnetization‐prepared rapid acquisition with gradient echo) images were acquired as follows: repetition time (TR)/echo time (TE), 1,900/2.52 ms; flip angle (FA), 9°; voxel size, 1.0 × 1.0 × 1.0 mm^3^; 300 slices; matrix, 256 × 256; field of view (FOV), 250 × 250 mm; acquisition time, 4 min 18 s.

For diffusion imaging, a 2D spin‐echo whole‐brain echo planar imaging pulse with the following parameter settings was used: TR/TE, 17,700/93 ms; FA, 90°; in‐plane resolution, 2.0 × 2.0 mm; 2.0‐mm effective slice thickness, 74 slices; matrix, 114 × 114; FOV, 224 × 224 mm. Thirty volumes with different gradient directions (*b* = 1,000 s/mm^2^) and two *b* = 0 volumes with reversed phase‐encoding (blip up/down) were acquired. Total scan time for the diffusion MRI was approximately 10 min.

Positron emission tomography scans were performed using a Siemens/Biograph TruePoint 16 Scanner (3D acquisition mode; 81 image planes; 16.2 cm axial FOV; 4.2 mm transaxial resolution; 4.7 mm axial resolution; 2 mm slice interval). A low‐dose CT scan was performed for attenuation correction before all scans. For tau imaging, participants were injected with 185 ± 37 MBq of ^18^F‐THK5351 prior to imaging and imaging was performed for a 20‐min PET acquisition, 40 ± 5 min post‐injection. For Aβ imaging, participants were injected with 555 ± 185 MBq of ^11^C‐PiB prior to imaging and imaging was performed for a 20‐min PET acquisition, 50 ± 5 min post‐injection. PET/CT data were reconstructed using an iterative 3D ordered‐subset expectation maximization reconstruction algorithm. The average interval between ^18^F‐THK5351, ^11^C‐PiB PET, and MRI was 20 ± 20 days (THK5351 to PiB = 12 ± 13 days; THK5351 to MRI = 12 ± 14 days; PiB to MRI = 17 ± 21 days).

### 2.3 MRI and PET data analyses

All images were preprocessed using the Statistical Parametric Mapping software (SPM12; Wellcome Department of Cognitive Neurology, London, UK) in MATLAB 7.12 (MathWorks, Natick, MA, USA). After partial volume correction using the PETPVE12 toolbox with the Müller–Gärtner approach (Gonzalez‐Escamilla et al., 2017; Müller‐Gärtner et al., 1992), both the ^11^C‐PiB and ^18^F‐THK5351 PET images were coregistered to the individual T1‐weighted images and anatomically standardized using DARTEL (Diffeomorphic Anatomical Registration Through Exponentiated Lie algebra; Ashburner, 2007).

After anatomical standardization, all PET images were normalized by the individual's positive mean uptake value in cerebellar gray matter. To investigate the anatomical features of cortical tau deposition that contribute to diffusion tensor tractography, we created volumes of interest consisting of the bilateral hippocampi, temporal lobes, anterior and posterior cingulate cortices, precunei, orbitofrontal lobes, and entire cerebrum using the Automated Anatomical Labeling atlas implemented in the Wake Forest University PickAtlas, version 2.4 (Maldjian, Laurienti, Kraft, & Burdette, 2003). ^18^F‐THK5351 accumulation in each region of interest was calculated as the standardized uptake value ratio using cerebellar gray matter as reference.

### 2.4 Diffusion tensor imaging data processing

Diffusion tensor imaging data were preprocessed using FMRIB Software Library, version 4.1 (Smith et al., 2004). Processing included eddy current correction and topup to correct for geometrical distortions and eddy currents. We used eddy software to detect and replace slices affected by signal loss due to bulk motion during diffusion encoding, which was performed within an integrated framework along with correction for susceptibility‐induced distortions, eddy currents, and subject motion (Jenkinson & Smith, 2001). Topup uses information from the reversed phase‐encoded blips, resulting in pairs with distortions in opposite directions. From these pairs, we estimated the susceptibility‐induced off‐resonance field and combined the two images into a single corrected image (Andersson, Skare, & Ashburner, 2003).

### 2.5 Diffusion MRI connectometry

Diffusion MRI connectometry was performed to explore white matter pathways that were related to tau deposition using DSI Studio (https://dsi-studio.labsolver.org; Yeh et al., 2016). The preprocessed DTI data of the CN group and AD‐spectrum group were reconstructed in a common stereotaxic space using q‐space diffeomorphic reconstruction to obtain the spin distribution function (SDF; Yeh & Tseng, 2011; Yeh, Wedeen, & Tseng, 2010). SDF values were used to estimate the local connectome and to construct a local connectome matrix. Then, correlation analysis was performed between the local connectome and six variables of preponderant tau deposition in AD (Sone et al., 2017). Six variables of tau deposition include tau in (a) bilateral hippocampi, (b) temporal lobes, (c) anterior and posterior cingulate cortices, (d) precunei, (e) orbitofrontal lobes, and (f) the entire cerebrum. We investigated which white matter pathways are correlated to each of the six variables of tau deposition. Six analyses were performed in the AD‐spectrum group, whereas another set of six analyses were performed in the CN group. This resulted in a total of 12 hypotheses examined using 12 connectometry analyses. For instance, the first connectometry analysis examined which white matter pathways in the AD‐spectrum group are correlated with tau deposition in bilateral hippocampi, with age as the covariate in the regression model. The second analysis examined the correlation with tau deposition in the temporal lobes using the same settings. The rest examined the correlation with tau deposition in the anterior and posterior cingulate cortices (third), precunei (fourth), orbitofrontal lobes (fifth), and entire cerebrum (sixth). A deterministic fiber tracking algorithm was used to connect local fiber directions with T‐scores >3, and tracts with connected lengths >40 mm were collected (Yeh, Verstynen, Wang, Fernández‐Miranda, & Tseng, 2013).

### 2.6 Statistical analyses

All statistical analyses were performed using SPSS software ver. 25.0 (SPSS Japan, Tokyo, Japan). Demographic variables were analyzed using Mann–Whitney test for continuous variables and the chi‐squared test for categorical variables between the AD‐spectrum group and the CN group. A *p*‐value <0.05 was considered statistically significant.

Using DSI Studio, we conducted a total of 2,000 randomized permutations to estimate false discovery rates (FDRs) of white matter pathways that exhibited increased or decreased connectivity related to tau deposition. To correct for multiple comparisons, FDR < 0.05 was considered significant.

## 3 RESULTS

### 3.1 Demographics

Participants’ demographic and clinical characteristics are presented in Table 1. No significant differences in age and sex were noted between the CN and AD‐spectrum groups. There was no significant difference in educational attainment between the groups. The CN group (global CDR = 0) showed an average MMSE of 29.3 ± 1.0, WMSR LM‐II of 12.3 ± 3.5, and MoCA‐J of 27.1 ± 2.4. The AD‐spectrum group showed an average MMSE of 22.0 ± 4.7, WMSR LM‐II of 2.4 ± 3.3, and MoCA‐J of 17.9 ± 4.9.

**Table 1:** Demographic characteristics of the participants

	CN group	AD‐spectrum group	
		aMCI	Mild AD
*N*	35	5	13
Age	66.4 ± 8.6	71.4 ± 8.5	68.0 ± 7.5
Sex (F/M)	20/15	2/3	10/3
Education	13.9 ± 2.2	14.2 ± 2.0	12.9 ± 2.4
CDR sum of boxes*	0.0 ± 0.3	1.4 ± 1.2	5.8 ± 2.5
CDR	0	0.5	1.0
MMSE*	29.3 ± 1.0	26.8 ± 4.4	20.2 ± 3.3
WMSR LM‐II*	12.3 ± 3.5	5.4 ± 4.6	1.2 ± 1.8
MoCA‐J*	27.1 ± 2.4	22.4 ± 5.2	16.2 ± 3.6

Note

CN: cognitively normal; AD: Alzheimer's disease; MCI: mild cognitive impairment;* N*: number of participants; F/M: female/male; CDR: Clinical Dementia Rating; MMSE: Mini‐Mental Status Examination; WMSR LM‐II: Wechsler Memory Scale‐Revised Logical Memory II MOCA‐J: Japanese version of the Montreal Cognitive Assessment.

Data are expressed as the mean ± standard deviation.

* Statistically significant (*p* < 0.05) between the AD‐spectrum group and the CN group.

### 3.2 Cortical accumulation of ^11^C‐PiB and ^18^F‐THK5351

**Figure 1:**
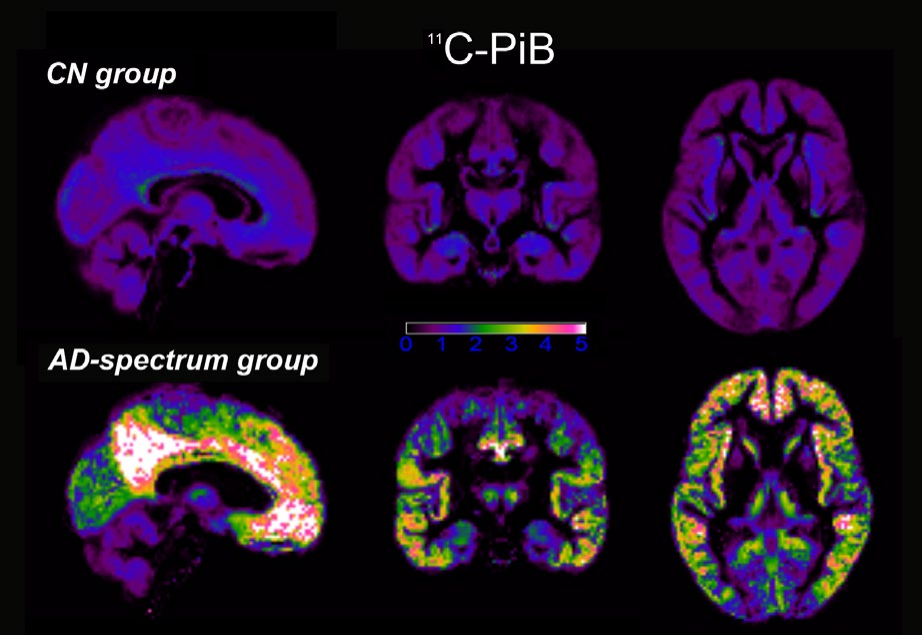
The AD‐spectrum group shows significantly increased uptake of ^11^C‐PiB on positron emission tomography. Amyloid accumulation is diffusely distributed throughout most of the neocortex, but particularly intense in the posterior cingulate cortex/precuneus, as well as the lateral temporal lobe and basal frontal lobe

All participants in the CN group were considered to be amyloid‐negative by visual assessment of ^11^C‐PiB, while all participants in the AD‐spectrum group were considered to be amyloid‐positive due to abundant tracer accumulation in the cerebral cortex. Although amyloid accumulation was diffusely distributed throughout most of the neocortex, it was particularly intense in the posterior cingulate cortex/precuneus, as well as in the lateral temporal lobe and basal frontal lobe (Figure 1).

**Figure 2:**
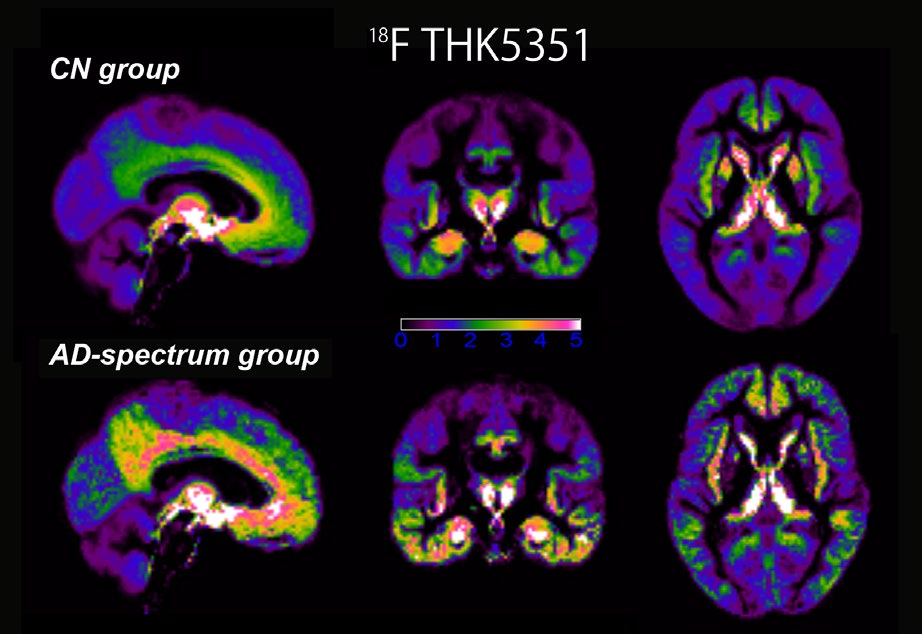
The amyloid‐negative CN group shows localized THK5351 accumulation particularly in the medial temporal lobe, extending to a lesser degree into the inferior temporal lobe, insula, posterior cingulate cortex/precuneus, and basal frontal lobe. The amyloid‐positive AD‐spectrum group shows higher tracer retention throughout temporal lobe, extending to widespread neocortical regions, most prominently in the posterior cingulate cortex/precuneus, and parietal and frontal lobe

In regard to tau accumulation, the CN group showed localized THK5351 accumulation particularly in the medial temporal lobe (entorhinal cortex, hippocampus, and parahippocampal gyrus), which extended to a lesser degree into the inferior temporal lobe, insula, posterior cingulate cortex/precuneus, and basal frontal lobe. The AD‐spectrum group showed higher tracer retention throughout the temporal lobe, which extended to widespread neocortical regions, most prominently in the posterior cingulate cortex/precuneus, and parietal and frontal lobe, but not the primary sensory and motor regions (Figure 2).

### 3.3 Diffusion MRI connectometry

**Figure 3:**
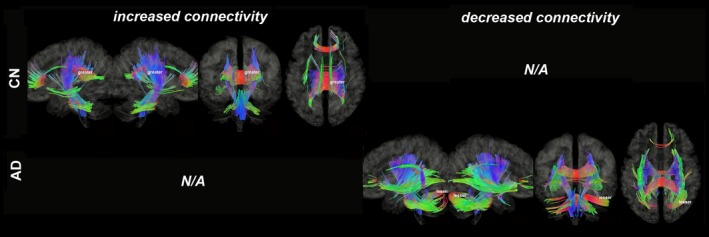
Structural connectivity correlated with tau deposition in bilateral hippocampus. The CN group shows increased connectivity (FDR = 0.0025), whereas the AD‐spectrum group shows decreased connectivity (FDR = 0). Red: left–right, green: anterior–posterior, blue: superior–inferior

**Figure 4:**
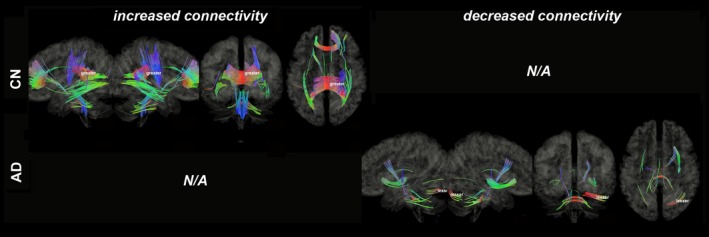
Structural connectivity correlated with tau deposition in temporal lobe. The CN group shows increased connectivity (FDR = 0.0023), whereas the AD‐spectrum group shows decreased connectivity (FDR = 0.015). Red: left–right, green: anterior–posterior, blue: superior–inferior

**Figure 5:**
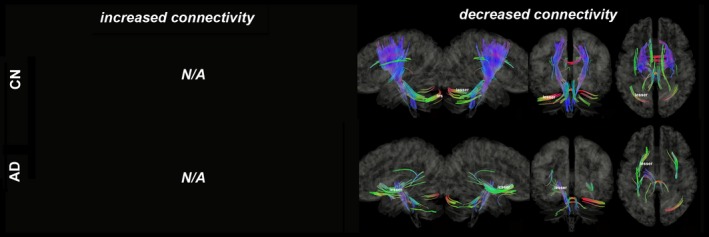
Structural connectivity correlated with tau deposition in anterior and posterior cingulate cortices. Both CN (FDR = 0.019) and AD‐spectrum groups show decreased connectivity (FDR = 0.024). Red: left–right, green: anterior–posterior, blue: superior–inferior

**Figure 6:**
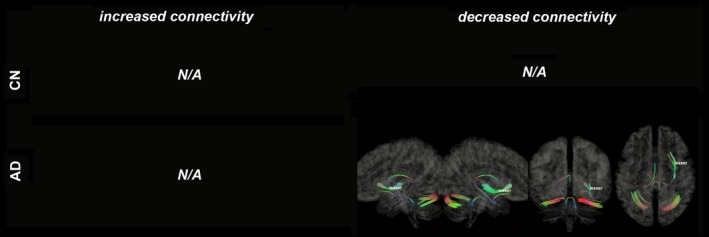
Structural connectivity correlated with tau deposition in precuneus. The CN group shows no significant connectivity, whereas the AD‐spectrum group shows decreased connectivity (FDR = 0.0074). Red: left–right, green: anterior–posterior, blue: superior–inferior

**Figure 7:**
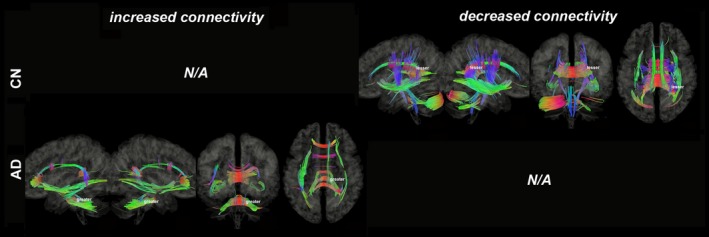
Structural connectivity correlated with tau deposition in orbitofrontal lobe. The CN group shows decreased connectivity (FDR = 0.0064), whereas the AD‐spectrum group shows increased connectivity (FDR = 0.0017). Red: left–right, green: anterior–posterior, blue: superior–inferior

**Figure 8:**
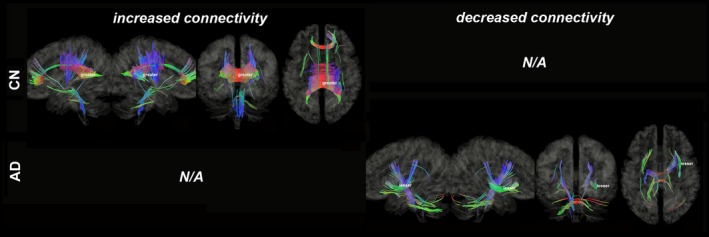
Structural connectivity correlated with tau deposition in entire cerebrum. The CN group shows increased connectivity (FDR = 0.0033), whereas the AD‐spectrum group shows decreased connectivity (FDR = 0.0129). Red: left–right, green: anterior–posterior, blue: superior–inferior

Figures 3, 4, 5, 6, 7, 8 show the connectometry results of increased and decreased connectivity correlated with ^18^F‐THK5351 deposition in the CN and the AD‐spectrum groups. Each image shows left sagittal, right sagittal, coronal, and axial views. The red, blue, and green colors indicate left–right, superior–inferior, and anterior–posterior orientations, respectively.

Structural connectivity correlated with tau deposition in the bilateral hippocampi (Figure 3) and showed increased connectivity of the corpus callosum (CC), inferior longitudinal fasciculus (ILF), cingulum, corticospinal tract (CST), medial lemniscus (ML), and superior/middle cerebellar peduncle (SCP/MCP; FDR = 0.0025) in the CN group, but showed decreased connectivity of the CC, superior longitudinal fasciculus (SLF), inferior fronto‐occipital fasciculus (IOF), uncinate fasciculus, CST, ML, SCP/MCP, and inferior cerebellar peduncle (ICP; FDR = 0) in the AD‐spectrum group.

Structural connectivity correlated with tau deposition in the temporal lobes (Figure 4) and showed increased connectivity of the CC, ILF, CST, and SCP/MCP (FDR = 0.0023) in the CN group, but showed decreased connectivity of the CC, IOF, CST, and MCP (FDR = 0.015) in the AD‐spectrum group.

Structural connectivity correlated with tau deposition in the anterior and posterior cingulate cortices (Figure 5) and revealed decreased connectivity of the CC, SLF, cingulum, CST, ML, and SCP/MCP (FDR = 0.019) in the CN group, and decreased connectivity of the ILF, IOF, CST, and MCP (FDR = 0.024) in the AD‐spectrum group.

Structural connectivity correlated with tau deposition in the precunei (Figure 6) and revealed no significant connectivity in the CN group, but showed decreased connectivity of the IOF and MCP (FDR = 0.0074) in the AD‐spectrum group.

Structural connectivity correlated with tau deposition in the orbitofrontal lobes (Figure 7) and revealed decreased connectivity of the CC, SLF/ILF, IOF, cingulum, CST, ML, and SCP/MCP (FDR = 0.0064) in the CN group, but increased connectivity of the CC, SLF/ILF, IOF, cingulum, and MCP (FDR = 0.0017) in the AD‐spectrum group.

In the entire cerebrum, structural connectivity correlated with tau deposition (Figure 8) and demonstrated increased connectivity of the CC, IOF, cingulum, CST, ML, and SCP/MCP (FDR = 0.0033) in the CN group, but with decreased connectivity of the IOF, CST, and MCP (FDR = 0.0129) in the AD‐spectrum group.

## 4 DISCUSSION

To our knowledge, this is the first study to focus on structural connectivity related to tau deposition in amyloid‐positive and amyloid‐negative groups using automated diffusion connectometry analysis. This analysis showed increased connectivity due to elevated tau deposition in the bilateral hippocampi, temporal lobes, and entire cerebrum in the CN group, but showed decreased connectivity in the bilateral hippocampi, temporal lobes, anterior and posterior cingulate cortices, precunei, and entire cerebrum in the AD‐spectrum group. These findings suggest that low levels of tau deposition without amyloid deposition induced compensatory responses against early neuronal injury or chronic inflammation associated with normal aging process. In contrast, the coexistence of amyloid with tau seemed to outweigh these compensatory effects, leading to decreased connectivity.

Neuropathological studies of cognitively healthy participants showed that NFTs were confined to the entorhinal and adjacent temporal lobe and were not often found in the extramedial temporal lobe or extratemporal cortex (Bouras, Hof, Giannakopoulos, Michel, & Morrison, 1994; Braak & Braak, 1991, 1997). Most previous tau PET studies also reported that tau deposition was confined to the medial temporal lobe. However, a recent large cohort study by Lowe et al. (2018) suggested that NFTs at advanced Braak stages (in the extramedial temporal lobe and extratemporal cortex) may be frequently detected in cognitively healthy individuals who are amyloid‐negative and noted that autopsy data supported their findings (Arriagada et al., 1992; Gertz et al., 1996). Our results are consistent with those of these previous studies: We detected tau outside the medial temporal lobe including in the inferior temporal lobe, insula, anterior and posterior cingulate cortices, precuneus, and orbitofrontal lobe (Braak stages III–IV), suggesting that primary age‐related tauopathy (PART)‐type pathology might be involved (Crary et al., 2014). Another possibility is that because of the characteristic binding of THK‐5351 to MAO‐B (Harada et al., 2018), elevated PET signals in the insula, and anterior and posterior cingulate cortices might be largely correlated to reactive astrocytosis (Carter et al., 2012). The AD‐spectrum group showed increased tau deposition in all isocortical areas, although the primary sensory, motor, and visual areas were spared, consistent with Braak stages V–VI.

We evaluated the correlation of structural connectivity with tau deposition in the bilateral hippocampi, temporal lobes, anterior and posterior cingulate cortices, precunei, and orbitofrontal lobes, in addition to the entire cerebrum. In the amyloid‐negative CN group with low tau deposition, we found increased connectivity due to tau deposition in the bilateral hippocampi, temporal lobes, and entire cerebrum. This is consistent with previous studies demonstrating increased functional connectivity in relation to higher tau tracer uptake, and can be explained as a compensatory response (Schultz et al., 2017; Sepulcre et al., 2017). We speculate that tau alone acts in a protective manner against early neuronal injury. However, the same argument does not necessarily hold for structural connectivity. An alternative explanation for increased connectivity in the CN group is chronic neuroinflammation associated with normal aging (Chung et al., 2009). Abbas et al. (2015) hypothesized that increased connectivity is a compensatory response to maintain normal cognition, giving the example of American football athletes in whom trauma caused neuroinflammation. Because ^18^F‐THK5351 retention reflects reactive astrocytes, which is one of the elements of neuroinflammation, glia activation or neuroinflammatory change may be more appropriate as the cause of increased connectivity in the CN group. Although neuroinflammation is a nonspecific finding, the combination of ^18^F‐THK5351 and structural connectivity might have potential for early diagnosis of preclinical AD if they could capture the alternating point of increased connectivity into decreased connectivity.

Meanwhile, the amyloid‐positive AD‐spectrum group showed decreased connectivity, which is consistent with previous studies that MCI/AD in general has often been associated with decreased structural connectivity (Mielke et al., 2012; Nowrangi et al., 2013; Wisse et al., 2015) and atrophy (Das et al., 2018; Maass et al., 2018; Xia et al., 2017). Aβ is hypothesized to increase tau deposition and to accelerate the spread of hyperphosphorylated tau beyond the collateral sulcus into the neocortex, via transsynaptic spread across neural networks. This spread leads to synaptic dysfunction and neuronal loss, resulting in cognitive decline (Liu et al., 2012; Price & Morris, 1999; Small and Duff, 2008). The AD‐spectrum group showed decreased connectivity despite a smaller difference in overall tau deposition compared with amyloid deposition, indicating that Aβ plays a critical role in connectivity responses. The neurotoxicity due to the coexistence of Aβ worsens with increasing levels of tau, and this counteracts any neuroprotective effects, leading to decreased connectivity in the AD‐spectrum group. It has been hypothesized that, if the coexistence of Aβ and tau leads to synaptic disruption and decreased connectivity, asymptomatic amyloid‐positive individuals corresponding to the first stage of preclinical AD without neuronal injury would show increased connectivity (Sperling et al., 2011). Further longitudinal studies including a cohort of preclinical AD patients are needed to test this hypothesis and clarify the interaction of Aβ and tau.

Interestingly, dissociation of increased connectivity in the CN group and decreased connectivity in the AD‐spectrum group was especially prominently associated with tau deposition in the hippocampus. The hippocampus plays a critical role in episodic memory and is a key component of the Papez circuit (Aggleton & Brown, 1999; Spiers, Burgess, Hartley, Vargha‐Khadem, & O'Keefe, 2001; Squire, 2004; Tsivilis et al., 2008). Neuroinflammation is also associated with normal aging, especially within the hippocampus (Hein & O'Banion, 2009); therefore, compensatory mechanisms associated with the hippocampus may be more significant compared with other brain regions, and when these mechanisms fail, there are likely to be more significant alterations in connectivity. Interestingly, we found almost the same result in the temporal lobe, although not as prominent as in the hippocampus.

The orbitofrontal lobe showed the opposite response to the hippocampus. Significantly decreased connectivity with elevated tau deposition was found in the CN group and may be the result of age‐related increases in atrophy levels in the orbitofrontal lobe as well as lower numbers of synapses (Fjell et al., 2014; Gogtay et al., 2004; Tamnes et al., 2013). In contrast, significantly increased connectivity with elevated tau was found in the AD‐spectrum group, and might be partly because cerebral blood flow and metabolism are preserved in the frontal lobe in the early stages of AD (Klunk et al., 2004; Matsuda, 2001). Another possibility may involve the differences in the distribution of mature and premature amyloid plaques. Two studies (Ikeda, Haga, & Kosaka, 1990; Yamaguchi, Nakazato, Shoji, Takatama, & Hirai, 1991) indicated that premature, diffuse amyloid plaques were present in the frontal lobe of AD patients, while more mature plaques were located in the temporal lobe; from these results, we hypothesized that mature plaques likely interacted with tau protein to produce negative effects.

This study has several limitations. First, there were small sample sizes for both AD patients and healthy controls, and preclinical AD patients were not included. Second, we did not collect genetic data, such as apolipoprotein E. Third, ^18^F‐THK5351 retention reflects reactive astrocytes in addition to tau pathology (Ng et al., 2017; Harada et al., 2018). Although we used the cerebellar cortex as the reference region, which is considered the least affected region of MAO‐B (Ng et al., 2017), and removed the age‐related influence of tau and MAO‐B (Braak, Thal, Ghebremedhin, & Tredici, 2011; Fowler et al., 1997), significant increased connectivity was found in the CN group. It can be said that tau and MAO‐B contribute to increased connectivity. However, whether each one or both were involved is unclear. Fourth, we did not define the threshold for ^18^F‐THK5351 to assess whether any of the control subjects had significant tracer uptake. We thought it difficult because it is impossible to collect the data of cognitively healthy young adults, so we could not compare the healthy older adults with young adults. Moreover, because both tau and MAO‐B increase with advancing age (Braak et al., 2011; Fowler et al., 1997), the proportion of tau and MAO‐B to ^18^F‐THK5351 retention is unknown.

## 5 CONCLUSIONS

Diffusion MRI connectometry detected significantly increased connectivity in the amyloid‐negative CN group with elevated tau deposition in the entire cerebrum, whereas decreased connectivity was found in the amyloid‐positive AD‐spectrum group. Low tau deposition seems to induce a compensatory response against early neuronal injury or chronic inflammation associated with normal aging, while the coexistence of amyloid and elevated tau seems to outweigh compensatory effects leading to decreased connectivity. Additionally, our results suggest that the coexistence of amyloid plays a crucial role in alternating the structural connectivity associated with tau deposition. Further longitudinal analysis that includes a cohort of preclinical AD patients is needed to assess the utility of the ^18^F‐THK5351 tracer.

## CONFLICT OF INTEREST

None declared.
